# Comparison between In Vitro Chemical and Ex Vivo Biological Assays to Evaluate Antioxidant Capacity of Botanical Extracts

**DOI:** 10.3390/antiox10071136

**Published:** 2021-07-17

**Authors:** Valentina Pasqualetti, Vittoria Locato, Chiara Fanali, Nadia Mulinacci, Sara Cimini, Anna Maria Morgia, Gabriella Pasqua, Laura De Gara

**Affiliations:** 1 Unit of Food Science and Nutrition, Department of Science and Technology for Humans and the Environment, Università Campus Bio-Medico di Roma, Via Álvaro del Portillo 21, 00128 Rome, Italy; v.pasqualetti@unicampus.it (V.P.); c.fanali@unicampus.it (C.F.); s.cimini@unicampus.it (S.C.); l.degara@unicampus.it (L.D.G.); 2 Pharmaceutical and Nutraceutical Section, Department of Neurosciences, Psychology, Drug Research and Child Health, University of Florence, Via Ugo Schiff 6, Sesto Fiorentino, 50019 Florence, Italy; nadia.mulinacci@unifi.it; 3 Department of Hematology, Stem Cell Transplantation, Transfusion Medicine and Cellular Therapy, Università Campus Bio-Medico di Roma, Via Álvaro del Portillo 21, 00128 Rome, Italy; a.morgia@unicampus.it; 4 Department of Environmental Biology, Universiy La Sapienza, Piazzle Aldo Moro 5, 00185 Rome, Italy; gabriella.pasqua@uniroma1.it

**Keywords:** human red blood cell membranes, polyphenols, phenolic acids, oxidative stress, lipid peroxidation

## Abstract

The anti-oxidative activity of plant-derived extracts is well-known and confers health-promoting effects on functional foods and food supplements. Aim of this work is to evaluate the capability of two different assays to predict the real biological antioxidant efficiency. At this purpose, extracts from five different plant-derived matrices and commercial purified phytochemicals were analyzed for their anti-oxidative properties by using well-standardized in vitro chemical method (TEAC) and an ex vivo biological assay. The biological assay, a cellular membrane system obtained from erythrocytes of healthy volunteers, is based on the capability of phytochemicals treatment to prevent membrane lipid peroxidation under oxidative stress by UV-B radiation. Plant extracts naturally rich in phenols with different structure and purified phytochemicals showed different in vitro and ex vivo antioxidant capacities. A high correlation between phenolic contents of the plant-derived extracts and their ability to prevent oxidative injuries in a biological system was found, thus underlying the relevance of this class of metabolites in preventing oxidative stress. On the other hand, a low correlation between the antioxidant capacities was shown between in vitro and ex vivo antioxidant assay. Moreover, data presented in this work show how food complex matrices are more effective in preventing oxidative damages at biological level than pure phytochemicals, even if for these latter, the antioxidant activity was generally higher than that observed for food complex matrices.

## 1. Introduction

Oxidative stress is a cellular condition induced by overproduction or ineffective removal of reactive oxygen species (ROS) that generally occurs in several diseases or responses to environmental stresses in all kinds of organisms, from microorganisms to humans. A certain amount of ROS production occurs even under physiological conditions, and it is even a sort of sensor able to properly trigger defense metabolic pathways [1]. However, when the endogenous ROS-scavenging mechanisms are insufficient or ROS production overcome a threshold value, an alteration of the physiological status of cell/tissue or organism is observed. So far, an always-increasing number of studies have demonstrated that oxidative stress is involved in many different pathological conditions such as cardiovascular diseases, diabetes, obesity, and cancer [2,3,4,5]. Several recent papers reported the importance of the uptake of foods rich in antioxidants, like vegetables, fruits, wine, tea, fruit juices, or food supplements with antioxidant properties to prevent the dangerous effects associated with oxidative stress [6,7,8,9,10,11,12,13]. In order to evaluate the potential health effects of such beneficial compounds, several different analytical methods have been developed and tested. Most of them have been reviewed by López-Alarcóna and Denicola [14]. A great multiplicity of assays is used for antioxidants testing. They differ in terms of experimental conditions, mechanisms by which the oxidant reaction is generated, the radical involved in the development of the signal etc. The most widely used tests are based on in vitro chemical tests, which exhibit positive as well as negative aspects. Generally, these antioxidant tests use free radical molecule traps and are based on colorimetric reactions. Among them, those ones so far used are free radical scavenging assays like the 2,2-diphenyl-1-picrylhydrazyl (DPPH) scavenging activity assay, the Trolox equivalent antioxidant capacity (TEAC) method, and the Ferric reducing-antioxidant power (FRAP) assay [3]. Although chemical assays are based upon well-known chemical reactions, these probably do not reflect the cellular physiological conditions.

Cultured cells have been often used to elucidate the mechanisms underlying the oxidative stress and to evaluate the protective effects of antioxidant molecules over different sources of oxidation. They give the possibility to study interactions between molecules/nutrients and cellular structures or metabolic pathways, thus providing results having higher biological significance. In the process of defining method requirements and standard operative conditions, the choice of cellular models represents an aspect that must be carefully considered. The properties and sensitivity of different cell lines are critical factors that significantly affect the evaluation of antioxidant activity. Indeed, an antioxidant is not only a substance able to prevent another substrate from oxidation, but a molecule that protects the whole biological system from damages coming from oxidizing stressors [15]. The advantage of using cultured cells resides in the fact that different stressors and cell types, including model systems for specific diseases, can be used for the evaluation of antioxidant effects. On the other hand, cell cultures have their own inconveniences: they are complex systems requiring a certain time for the analysis and the results could strictly depend on the used cell line. For example, widely used cell lines derive from cancer tissue or are the results of “immortalization” processes that can alter the physiological metabolism or the metabolic responses to a certain stimulus in comparison with a healthy cell. The presence of a culture medium, rich in ions and growth-regulating factors, is another critical point for the use of cell culture for evaluating the antioxidant capacity of a certain molecule, since components of culture medium could themselves interact with the antioxidant activity.

Another approach is the use of ex vivo cells. Due to their susceptibility to oxidation, and the facility of getting them, erythrocytes or red blood cells (RBCs) have been reported in several papers as model system to study the antioxidant protection by natural molecules against in vitro induced oxidative stress [2,16,17,18]. Therefore, RBCs may represent an interesting and biologically relevant model for a cell-based bioassay. The high membrane concentration of polyunsaturated fatty acids and the O_2_ transport, associated with redox active hemoglobin molecules, make erythrocytes relevant targets of free radical attack. Most of these methods use intact erythrocytes, evaluating different indicators of induced cellular stress damage like levels of membrane lipid peroxidation products, redox enzymes, protein oxidation products or grade of hemolysis, and osmotic fragility [19]. Nevertheless, cellular models are yet excessively complex to identify the types and the concentration of substrates that are the first direct targets within the intricate antioxidant cascade propagation. Plasma membrane is the first barrier and protection of the cells against external physical, chemical, or biological stimuli among which many stressors inducing ROS production. Many studies demonstrated that lipids, such as polyunsaturated phospholipids, are vulnerable target of free radicals, and that secondary products of lipid peroxidation induce further modification of biologically essential molecules [20,21,22]. Hence, plasma membrane might be considered a key tool for the control of the oxidative cascade triggering.

Focus of this work was the comparison of a well-standardized in vitro chemical method (TEAC) and an ex vivo biological assay based on erythrocytes membranes to predict the biological anti-oxidative capacity of four different phenolic plant-derived extracts naturally rich in phenols characterized by a different chemical structure [23], one food supplement and seven purified phytochemicals.

To have a broad representativeness of bioactive compounds with different chemical structures and antioxidant capability, extracts characterized by the presence of several classes of phenolic compounds and flavonoids have been selected. The protective effect of plant extracts and purified phytochemicals toward UV-dependent lipid peroxidation was also compared.

## 2. Materials and Methods

### 2.1. Materials and Reagents

All solvents were of HPLC grade and were used as received. Methanol and water were purchased from Farmitalia-Carlo Erba (Milan, Italy) and Sigma-Aldrich (Milan, Italy), respectively.

Sodium and potassium chloride, phenolic acid standard compounds (gallic acid ≥ 98%, caffeic acid ≥ 99%, vanillic acid ≥ 97%, protocatechuic acid ≥ 98%, resveratrol ≥ 99%, ellagic acid ≥ 99%), 2-thiobarbituric acid, 1,1,3,3-tetramethoxypropane (MDA) and (±)-6-hydroxy-2,5,7,8-tetramethylchromane-2-carboxylic acid (Trolox), ascorbate (≥99%), and gluthatione (≥98%) were from Sigma-Aldrich (Milan, Italy). Cellulose acetate membrane filters (0.2 μm) were purchased from Sartorius Stedim Biotech S.A (Aubagne Cedex, France).

### 2.2. Plant Extracts

Plant-derived extracts tested in this study were obtained from plant food, plant by-products from the agro-food industry derived from wine and oil agro-industry and dietary supplements. In particular: seeds from grapes (*V. vinifera* L. cv. Italia) (ITA-seeds); leaves of rosemary (*Rosmarinus officinalis* L.) (ROSE-leaves); mesocarp of pomegranate fruits (*Punica granatum* L. cv. Wonderful) (POM-mesocarp); OLIVE-water obtained from filtered vegetation water from olive milling and Vineatrol^®^30, a commercial food supplement (Breko GmbH, Bremen, Germany). All the samples were extracted according to Mulinacci et al. [23] and were solubilized in ethanol/water (1:1, *v*/*v*) solvent at concentration 5 mg/mL, 20 mg/mL, 10 mg/mL, 10 mg/mL, and 40 mg/mL for ITA-seeds, VINEATROL, ROSE-leaves, POM-mesocarp, and OLIVE-water, respectively. The extracts have been freeze-dried.

### 2.3. Antioxidant Standard Solutions

Gallic acid, caffeic acid, vanillic acid, protocatechuic acid, and resveratrol were dissolved in methanol at the concentration of 50 mM (stock solutions) and were stored at −80 °C. Ellagic acid was dissolved in 1 M NaOH at the concentration of 30 mM. To perform analyses, phenolic acid mixtures were prepared diluting stock standard solutions with distilled water at the final concentrations of 10, 50, 100, 125, 250, and 500 µM (working solutions).

Two well-known antioxidants, ascorbate (ASC) and glutathione (GSH), were also tested. ASC and GSH standard were solubilized in aqueous NaCl 0.9% (*w*/*v*) solution.

For each sample, solutions at different concentrations were tested in order to identify the range within which the protective effect followed a linear trend by avoiding saturation effects of their protective capacity toward biological membranes and for identifying the concentration of the antioxidant/extract able to give a 50% protection of the imposed oxidative stress.

### 2.4. Determination of Plant Extracts Total Phenolic Compounds

Total phenolic content (TPC) was assayed according to the Folin–Ciocalteau method [24], with a little modification, using gallic acid as standard. A volume of 1580 μL of distilled water and 100 μL of Folin–Ciocalteu reagent were added to 20 μL of sample extract (or standard solution) and incubated at room temperature for 8 min. Then, 300 μL of sodium carbonate solution (20% *w*/*v*) was added. The solution mixture was incubated in the dark at room temperature for two hours. The absorbance at 765 nm was measured in a 96-well plate (Greiner Bio-one, Frickenhausen, Germany) using a multifunctional microplate reader (Tecan Infinite 200 PRO multiplate reader, Männedorf, Switzerland). The estimation of TPC in the samples was calculated by a calibration curve obtained with gallic acid. The results are expressed as mg of gallic acid equivalents (GAE) per g of dry weight (DW) sample.

### 2.5. Determination of Plant Extracts Radical-Scavenging Capacity by ABTS/TEAC Assay

The ABTS radical scavenging activity was assessed according to the method described previously [25] with minor modifications. The radical cation ABTS^•+^ was produced by reacting 7 mM ABTS aqueous phosphate buffer (5 mM NaH_2_PO_4_-H_2_O and 5 mM Na_2_HPO_4_-2H_2_O, pH 7.4) solution with 2.5 mM potassium persulfate (final concentration). The mixture stands for 12 h at room temperature and in the dark for reaction. Prior to use in the assay, ABTS^•+^ working solution was obtained diluting in aqueous phosphate buffer stock solution to an absorbance of 0.70 ± 0.05 at 734 nm. A volume of 10 μL of diluted extract (or purified phytochemicals) was mixed with 190 μL of ABTS^•+^ working solution in a 96-multiwell plate and the absorbance was recorded at 734 nm after 20 min. A calibration curve was prepared with Trolox as a standard used in a concentration range 0–700 μM. Results are expressed as μmol Trolox equivalent (TE) per mg of sample.

### 2.6. Red blood Cell Membranes Preparation

Human venous blood from different healthy volunteers was obtained by venipuncture and collected in tubes containing heparin. Healthy donors (men and women aged between 18 and 65 years) were granted by Transfusion Center of Campus Bio-Medico University Hospital in Rome, after authorization of the department’s manager and appropriate signed and informed consent by involved patients. Blood samples were collected within 48 h before experiment. Blood samples were centrifuged at 3500× *g* for 5 min at 4 °C (Megafuge 1.0 R Heraeus centrifuge, San Diego, CA, USA) to allow separation of plasma from RBCs. After the removal of plasma and buffy coat, packed RBCs were carefully recovered from the bottom of the vial. To reduce inter-individual variability, a pool of collected packed RBCs was prepared from 4–6 donors’ blood. RBCs were then stored at 4 °C until their utilization for biological assays. The criteria for the selection of blood samples were the number of red blood cells (suitable range, 4.5–5.0 × 10^6^/μL) and packed cell volume (suitable range, 39–45%).

Hemoglobin-free RBCs membranes were prepared according to Cavallini et al. [26] with minor modifications. Briefly, a volume of 500 µL of packed RBCs was mixed with 9 mL of ice-cool distilled water and incubated in ice for 30 min to obtain complete hypotonic lysis of the erythrocytes. About 1 mL of KCl (2 M) was then added and the mixture was incubated for an additional 5 min. Total of 1 mL of the membrane suspension (about 8 × 10^8^ ghosts, corresponding to about 50 mg of membranes) was filtered on a 0.2 μm cellulose acetate membrane filter (11107-50-CAN, Sartorius Stedim Biotech S.A Aubagne Cedex, France) under vacuum. The thin uniform layer of RBCs membranes was washed using 0.9% (*w*/*v*) NaCl water solution, until dried colorless membranes were obtained.

### 2.7. Oxidative Stress Induction

The effects of antioxidative molecules on protection against RBCs membranes UV-oxidative stress were investigated as follows. Cellulose acetate membrane filter containing RBCs membranes were incubated for 60 s under slight agitation with 1 mL of solution containing different concentrations of antioxidant metabolites, the range of concentrations was chosen in order to reach a plateau in the protection against the oxidative injury. After 60 s incubation, filters were washed, dried under vacuum, and finally placed into Petri dishes containing 4 mL of 0.9% (*w*/*v*) NaCl solution.

RBCs membranes layered on cellulose acetate filters were than irradiated with UV-B for 40 min. A parallel bank of two UVB-313 fluorescence tubes (Gold Light Power Intensive 80W SR, UV Type 2, 22,210,411 CE-H308), emitting a continuous spectrum between 280 and 320 nm, was used for irradiation. UV-B irradiance was measured having an average intensity of 3850 Lux with a typical irradiance in bed of 200 W/m^2^ as reported on product’s technical data sheet. Light irradiance was measured by means of a united detector technology radiometer (UDT instruments, San Diego, CA, USA). At the end of UV-B radiation time, 200 μL of solution was collected from each Petri dish for the determination of MDA concentration.

The following samples were analyzed:

- A negative control corresponding to RBCs membranes sample kept in the dark, untreated with plant extract and not exposed to UV-B irradiation;
- A positive control corresponding to RBCs membranes sample untreated with plant extract and exposed to UV-B irradiation. It represents the maximum inducible stress (i.e., maximum MDA levels produced after irradiation in the absence of protective substances);
- Treated samples corresponding to RBCs membranes incubated with plant extracts or phytochemical solution and exposed to UV-B irradiation.

### 2.8. Determination of MDA Level

Malondialdeyde, which was release by the erythrocytes membranes as a product of lipid peroxidation, was determined by the thiobarbituric acid reactive substances (TBARS) assay [27]. In order to identify the most appropriate time for the analysis, the MDA released from erythrocyte membranes has been measured during UV exposure time ranging between 5 and 60 min. Such production shows a non-linear increment of MDA levels that might be described with good approximation (R^2^ ≈ 0.99) by a quadratic polynomial function (Figure S1). On the basis of the obtained results, 40 min has been considered the good medium between time for the analysis and MDA production adequate for making evident the protective effect of the added antioxidants. Therefore, this time has been selected to perform all the experiments.

A volume of 200 μL of sample or MDA standard solution was added to the same volume of 0.67% (*w*/*v*) TBA solution and the mixture was heated at 90 °C for 40 min. Then the solution was cooled and immediately analyzed. Spectrofluorimetric determination of the MDA(TBA)_2_ adduct was performed on infinite 200 PRO instrument (Tecan, Männedorf, Switzerland) using the i-control software for data acquisition. An opaque 96-well black flat bottom polystyrene plate (Corning Costar, New York, USA) was loaded with a volume of 200 μL of samples or MDA(TBA)_2_ standard solution per well. Fluorescence was measured, setting the excitation and emission wavelengths at 515 nm and 550 nm, respectively. An MDA standard calibration curve was built using an MDA concentration range of 0.01–1 μM.

The values of MDA obtained in the negative control was subtracted to the MDA values obtained in the positive control and treated sample in order to avoid the interference of the different blood batches and the oxidative stress not depending to the UVB treatment. The protective effect was expressed as percentage inhibition of MDA formation with respect to the irradiated positive control samples according to the following formula:

(1) $$Protection\;\left(\%\right)=\left(1-\frac{\mathrm{MDA}\;sample}{\mathrm{MDA}\;control}\right)\times 100$$

In the formula, MDA *sample* is referred to the concentration of MDA in RBC membranes samples pre-treated with plant extracts or phytochemical solutions after subtraction of MDH of the negative control; MDA *control* is the concentration of MDA in irradiated RBC membranes samples without any pre-treatment, after subtraction of the negative control. All analyses were done in triplicate and the results expressed as mean values ± SD.

The protective capacity of the different samples was also expressed as IC_50_ (IC = inhibiting concentration), i.e., as sample concentration (in µg/mL) that determines 50% of inhibition of MDA release induced by radiation stress. For plant samples and antioxidant standards, the IC_50_ was calculated by replacing the y value of the equation curve obtained at the analyzed concentration (see Results) with 50% and by resolving the mathematical equation with respect to × value.

### 2.9. Statistical Analysis

All samples were analyzed, replicated three times, and the results are presented as mean ± S.D. Descriptive statistical analyses were performed using Excel software (Microsoft Office 2002) for calculating the means and the standard error of the mean. Statistical significance was calculated with analysis of variance (ANOVA) that was performed by means of GraphPad Prism statistical software program (4.02 version; San Diego, CA, USA) using a variance’s analysis of one-way modality and non-parametric tests followed by Tukey’s multiple comparison test. Differences with *p*-value less of 0.05 (*p* < 0.05) have been considered to be statistically significant and have been marked by different letters in the figures.

## 3. Results and Discussion

**Figure 1:**
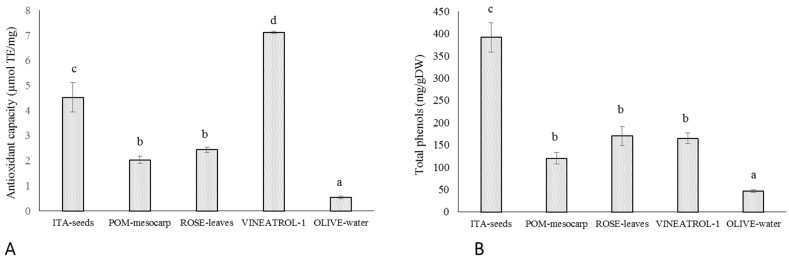
Antioxidant features of plant food-derived matrices and a commercial food supplement. (**A**). The antioxidant capability of extracts from ITA-seeds (seeds from *V. vinifera* L. cv. Italia), ROSE-leaves (leaves from *Rosmarinus officinalis* L.) POM-mesocarp (mesocarp of *Punica granatum* L. cv. Wonderful fruits) OLIVE-water (water obtained from olive milling), VINEATROL-1 (a food supplement from Breko GmbH, Bremen, Germany) was determined as ABTS scavenging activity by TEAC assay. Results are expressed as Trolox equivalent µmols per mg of dry sample. (**B**). The total content of phenols of the same samples have been determined by Folin–Ciocalteau method and expresses as mg per dry weight g of sample. Different letters (a), (b), (c), (d) indicate a statistical difference (*p* < 0.05) according to ANOVA.

In this study, the in vitro radical scavenging capacity of selected plant extracts was determined by ABTS/TEAC assay (see Material and Methods for details). It is a widely used in vitro chemical test for measuring the total antioxidant capacity of food and nutraceuticals [28,29]. As it is shown in Figure 1A, Vineatrol shows the highest antioxidant capability, followed by ITA-seeds. POM-mesocarp and ROSE-leaves show intermediate and comparable antioxidant capability, whereas OLIVE-water has the lowest antioxidant capability. The total phenolic content of the extracts was also determined (Figure 1B). ITA-seeds show the highest phenolic content (approximately 40% of DW), followed by ROSE-leaves, Vineatrol, POM-mesocarp, and OLIVE-water (below 20% of DW) (Figure 1B). The chemical characterization, in term of polyphenolic composition, of the plant-derived extracts used in this study is described by Mulinacci et al. [23]. They derive from plant-derived food or food chain by-products and contain different mixture of antioxidant compounds being principally phenolic compounds together to vitamin C, in some of them (POM-mesocarp, ROSE-leaves and Vineatrol). OLIVE-water sample, a by-product derived from olive oil production, contains simple phenols, in particular hydroxytyrosol, a well-known compound with a great number of demonstrated human health beneficial effects [30]. POM-mesocarp sample, an extract from the peel of pomegranate fruit, is an important by-product deriving from juice production [31]. They are rich of a specific class of phenolic compounds called ellagitannins. In particular, two isomers called punicalagins characterize pomegranate peel and are used in the nutraceutical field [31,32]. The extract of rosemary leaves (ROSE-leaves) is characterized by the presence of carnosic and rosmarinic acids, both recognized as potent antioxidants but also anti-inflammatory, anti-nociceptive, and hepatoprotective agents [33]. POM-mesocarp and ROSE-leaves also contain 1.5 and 0.5 mg/g of ASC, respectively [23]. Grape seed (ITA-seed) extracts have been widely studied for their health effects and are already used as components of food supplements. ITA-seed is rich in monomeric flavan-3-ols as (+)-catechin, (−)-epicatechin and (−)-epicatechin-3-O-gallate, as well as in dimeric and polymeric procyanidins. A commercial food supplement, Vineatrol^®^, was also used in this study. This extract contains the stilbenes resveratrol and viniferin, as well as a small amount of ASC (0.22 mg/g), [23]. Noteworthy is that the concentration of ASC in POM-mesocarp, ROSE-leaves and Vineatrol is very low thus suggesting a negligible contribute of this vitamin to the antioxidant effect.

These results confirm that in vitro tested antioxidant capability depends on both type and amount of the antioxidant molecules present. Indeed, OLIVE-water, that contains the lowest amount of polyphenols, also has the lowest antioxidant capacity, POM-mesocarp and ROSE-leaves having similar amount of polyphenols also have similar antioxidant capacity. On the other hand, in ITA-seeds and Vineatrol the total phenolic amount did not mirror the extracts’ antioxidant capability, since Vineatrol has an antioxidant capacity, chemically measured, about 40% higher than ITA-seeds despite its phenolic content is less than half than that of ITA-seed.

Being many human diseases, such as neurodegenerative disorders, cardiovascular diseases and cancer, correlated to oxidative stress [34,35,36], the protective effect of antioxidant supplements in pathological framework has been also widely discussed in literature. At this concern, many clinical studies revealed that oxidative injuries were not significantly reduced by supplementation of single or combination of few antioxidants, mainly vitamin E, vitamin C, and carotenoids (reviewed by Villanueva and Kross, [37]). Diversely, the use of plant extracts naturally rich in bioactive molecules seems to be more efficient in preventing oxidative damages [38]. This can depend on the fact that different antioxidant molecules effectively work in a synergic fashion when they are simultaneously present. In fact, different antioxidant redox couples work in a network where the oxidized form of a redox molecule is converted back into the biological active one (reduced form) by another redox couple with a lower redox potential [37]. For this reason, in this study the efficacy of plant extracts in reducing cellular oxidative damage was also compared to the protective effect of known antioxidants, commercial standards of phenols and Vineatrol, a commercial food supplement.

Therefore, the antioxidant capacities of purified phenols and well-known antioxidants, such as ascorbate (ASC) and glutathione (GSH), largely represented in plant food, have been also measured with TEAC in in vitro chemical assay. These metabolites exhibit radical-scavenging activities in the following order: gallic acid > caffeic acid ≥ resveratrol ≥ ellagic acid ≥ glutathione> protocatechuic acid ≥ vanillic acid > ascorbic acid (Table 1). The difference in antioxidant properties of the phenolic molecules are in accordance with the literature [39]. It is interesting to notice that this capacity greatly varies in the analyzed phenolic standards.

It is well-known that in vitro chemical assays have several limitations in the study of molecules with antioxidant properties, mainly because they do not consider relevant parameters involved in biological systems. Moreover, an antioxidant able to counteract oxidative damages in a biological context must be able to directly scavenge overproduced ROS but also avoid or revert ROS-derived damages of biological macromolecules. In order to study the biological protective effect of the various extracts or phytochemicals, a model of oxidative stress induced by UV-B radiation in human red blood cells membranes has been set up.

The oxidative damage due to UV-B radiation has been monitored in terms of increased release of malondialdehyde (MDA) from a suspension of isolate RBCs membranes as a consequence of lipid peroxidation. The ability of the different extracts to prevent membrane oxidative damage has been evaluated by using this experimental approach. This experimental model has different advantages: (i) It gives information on a real biological situation; (ii) the use of RBC membranes is a simple and homogeneous human model and, since the membrane are pooled from different volunteers, the effect of individual variability is limited; (iii) the obtainment of RBC membranes does not require a laboratory-intensive practice, diversely from the isolation of other biological systems used at this purpose in the literature, such as microsomes, blood LDL or mammal tissues, the latter also requiring lab animal sacrifice; (iv) in comparison with cell cultures, it allows to avoid the interference with possible oxidative catalysts present in the cell culture medium, such as transition metals, and therefore, chemical interferences affecting the accuracy of the assay are avoided. The imposition of an oxidative stress by a physical method, such as UV-B radiation, instead of by a chemical oxidant also has its advantage, since it avoids the possible direct interference between the stressor and the antioxidant molecule or mix of molecules that are under study. For example, metal ions are known to induce an oxidative stress in biological system, but they could be directly chelated or blocked in their oxidative reaction by specific organic molecules.

**Figure 2:**
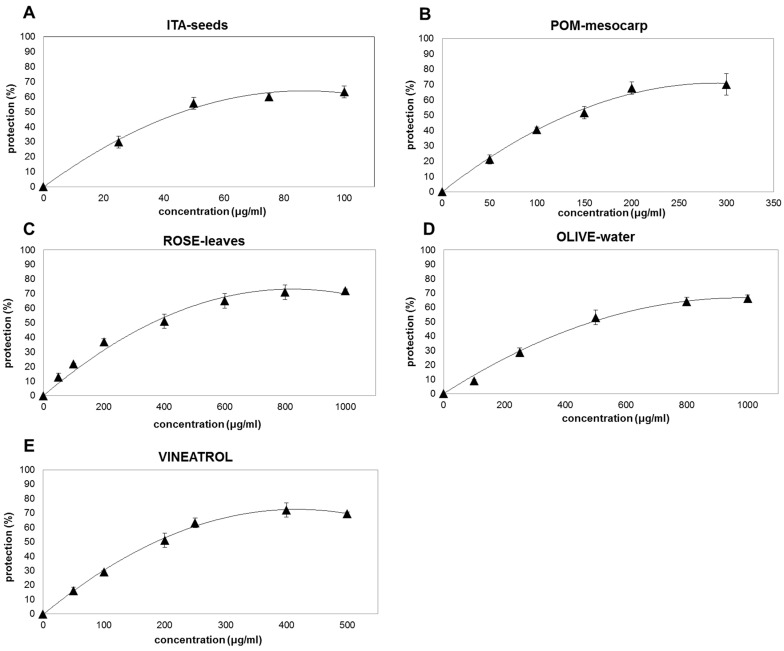
Protective effect of extracts from plant food-derived matrices and a commercial supplement on RBC membrane oxidation by UV-B radiation. The protective effects of different phenolic plant extracts (ITA-seeds (**A**); POM-mesocarp (**B**); ROSE-leaves (**C**); OLIVE-water (**D**); VINEATROL (**E**)) are expressed as percentage inhibition of MDA formation induced by UV irradiation in the RBC membranes pre-incubation with different concentration of the antioxidants in comparison with the MDA produced by the same RBC membrane in absence of the same antioxidant mixes or purified molecules. The concentration of the antioxidants was increased until reaching a plateau indicating that saturation in the protective efficiency has been reached. The values are the means of three independent experiments ± SD.

**Figure 3:**
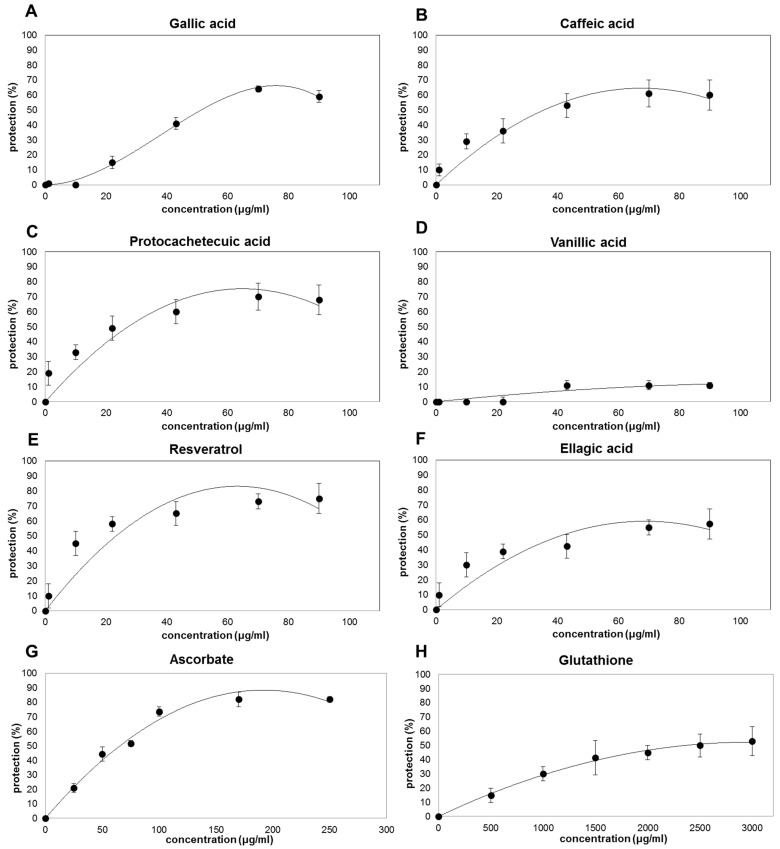
Protective effect of purified standard molecules on RBC membrane oxidation by UV-B radiation. The protective effects of different antioxidant standard molecules (Gallic Acid (**A**); Caffeic Acid (**B**); Protocachetecuic Acid (**C**); Vanillic Acid (**D**); Resveratrol (**E**); Ellagic Acid (**F**); Ascorbate (**G**); Glutathione (**H**)) are expressed as percentage inhibition of MDA formation induced by UV irradiation in the RBC membranes pre-incubation with different concentrations of the antioxidants in comparison with the MDA produced by the same RBC membrane in absence of the same antioxidant mixes or purified molecules. The concentration of the antioxidants was increased until reaching a plateau indicating that saturation in the protective efficiency has been reached. The values are the means of three independent experiments ± SD.

When RBCs membranes are pre-incubated with plant extracts or purified standards before UV-B-exposition, a dose-dependent decrease in MDA production is clearly observed, with the exception of vanillic acid that is almost ineffective (Figure 2 and Figure 3). It is interesting to notice that, despite the differences in their antioxidant capacity observed by ABTS/TEAC assay (Figure 1A, Table 1), all plant extracts and the active phenolic phytochemicals protect up to about 60–70% of the oxidative injury (Figure 2 and Figure 3). However, different concentrations are necessary to reach the maximum protective effect (between 100 and 800 μg/mL for plant-extracts (Figure 2) and between 50 and 100 μg/mL for the purified phenolic standards resveratrol, gallic acid, caffeic acid, and protocatechuic acid (Figure 3A–F)). GSH has a protective effect of only 50% at concentration of 3000 μg/mL (Figure 3H); while ASC seems to be the most effective in protecting erythrocyte membranes showing a decrease in the oxidative damages of about 80% at the concentration of about 250 μg/mL (Figure 3G). This result is consistent with the capacity of ASC to protect cellular membrane, in spite of its hydrophilic nature [40].

In order to have other information on the protective effects of the different molecules or mix of molecules, the values of concentrations able to induce a 50% reduction in the MDA production (IC_50_) has been calculated (Table 2). Among the extracts, ITA-seeds have the lowest IC_50_ highest protective effect against oxidative damage induced by UV-B radiation on the erythrocytes membranes (IC_50_ of 39.5 ± 6.8 µg/mL). Such protection would appear to be similar to that of caffeic acid and ellagic acid (IC_50_ of 44 ± 11 and 45 ± 10 μg/mL, respectively); whereas gallic acid and ASC showed a slightly above IC_50_ (64 ± 10 and 60 ± 7 μg/mL, respectively). The highest protective effect was shown for resveratrol (IC_50_ of 12 ± 8 µg/mL); while the lowest protective capacity was observed for GSH and OLIVE-water (IC_50_ of 2226 ± 172.3 and 397± 66.4 μg/mL, respectively). The extracts of pomegranate (POM-mesocarp), rosemary (ROSE-leaves), and the extracted food supplement Vineatrol showed intermediate protection (IC_50_ of 155 ± 18, 169 ± 4.9 and 193 ± 1.1 μg/mL, respectively). It is important to note that the protective effect of the analyzed extracts against oxidative damage determined in vivo at cellular level is not directly related to the total antioxidant capacity of the same extracts measured in vitro with chemical assays. In particular, for the extracts, a greater correlation seemed to be present between IC_50_ and the total phenolic content (Figure 1B and Table 2). For example, ITA-seeds (having the highest content of total phenols) showed the lowest IC_50_; on the contrary, OLIVE-water (having very low phenolic content) showed the highest IC_50_. Intermediate levels of total phenols (as observed for pomegranate, rosemary, and Vineatrol extracts) correspond to intermediate protective effects. The knowledge of phenolic concentration ranges effective for preventing oxidative damages is of interest due to the fact that these metabolites are only marginal components of plant tissues and usually have modest bio-availability [41,42]. Noteworthy is that the total phenolic content in the extracts is below 20% of their dry weights (Figure 1B) with the only exception of ITA-seeds which reaches approximately 40%. Moreover, the very low concentration of ASC in POM-mesocarp, ROSE-leaves, and Vineatrol, suggests a negligible contribution of this vitamin to the antioxidant effect. Taking into account these considerations and comparing the IC_50_ values in Table 2 between the pure molecules and the tested extracts, it can be hypothesized that the phenolic pools of the plant extracts, which constitute the phytocomplex, are generally more effective than the single pure molecules.

Our results also suggest that the various plant phenolic molecules interact differently with erythrocytes’ phospholipid membranes as electron donors. This would increase the function of antioxidant redox systems of red blood cells by carrying out a biologically relevant radical-scavenging activity. In particular, by comparing ITA-seeds and Vineatrol it is possible to observe how the commercial compound, despite its highest antioxidant capacity (7.1 ± 0.4 µmol TE/mg; Figure 1A), is required at higher concentration for being effective (IC_50_ of 193 ± 1.1 μg/mL; Table 2) than grapes seeds having lower antioxidant capacity (4.5 ± 0.6 nmol TE/mg and IC_50_ of 39.5 ± 6.8 μg/mL; Figure 1A; Table 2). The slightest protective effect exhibited by Vineatrol might probably be the consequence of its more limited composition, in terms of diversity of phenolic compounds, than that of grape seeds. Indeed, a polyphenolic content of 392 mg/g was found in ITA-seeds, of which about 90% were different polymers with a degree of polymerization ≥4 (Pol1 + Pol2 both as non-acylated forms and as acylated forms with gallic acid residues) and the remaining 10% other phenolic compounds (procyanidins, catechins, and epicatechins [23]. Vineatrol, on the other hand, is a mixture of only three stilbenes: resveratrol, ε-viniferin, and δ-viniferin, all characterized by the absence of catechol groups in their chemical structure. On the other side, the more heterogeneous mix of molecules present in ITA-seed extract, which present several catechol residues, could be responsible for the better efficacy in protecting a biological system from oxidative stress despite its relatively lower chemically measured antioxidant capability.

**Figure 4:**
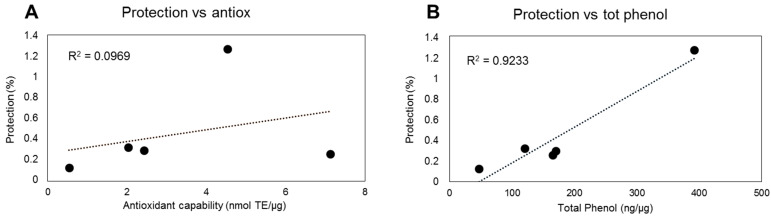
Correlation between protective effect of extracts from plant food-derived matrices and their antioxidant capability (**A**) and total phenol content (**B**). The protective effect of different phenolic plant extracts (1 µg) was correlated with their antioxidant capability or total phenol content.

In relation to the phenolic composition of the plant-derived extracts, the obtained results indicate a positive correlation between phenols’ content and heterogeneity and the efficacy in preventive oxidative damage, in terms of lipoperoxidation (Figure 4B). These results further underlie the relevance of this class of metabolites in preventing oxidative stress. On the other hand, the correlation between chemical antioxidant capacity and efficacy in protecting against oxidative damage in a biological system seems to be less significant (Figure 4A).

In relation to the protective capacity of the phenolic purified standards, the results related to vanillic acid are rather surprising. This phenol had a chemically measured antioxidant capacity in line with that of the other analyzed molecules (Table 1); however, it was almost inefficient in protecting RBC membranes from the UV-derived oxidative damages (Table 2). This result suggests that the catechol group, present in the other phenolic acids, but absent in the structure of vanillic acid, is crucial to exert a protective effect in RBC membranes. The antioxidant capability of phenolic acids has been mainly referred to their capability to donate hydrogen atoms. In general, substituents on the aromatic ring, numbers, and positions of the hydroxyl groups in relation to the carboxyl functional group, esterification, glycosylation seem to affect the antioxidant ability. Generally, higher OH number corresponds to higher antioxidant ability and hydroxylated cinnamates are more effective than benzoate counterparts in donating hydrogen atom [43]. Consistently, gallic acid showed the highest antioxidant capacity (Table 1), but the in vitro chemical antioxidant capability did not mirror the biological antioxidant protective effect of this compound (Table 2), underlining the importance to evaluate the in vivo effect of redox metabolites. Moreover, the obtained results show that pure antioxidants showing comparable IC_50_, such as gallic acid, caffeic acid, ellagic acid, and ascorbate, differ in terms of in vitro antioxidant capability (Table 1 and Table 2). This underlines that their protective mechanism depends on the biological context where they work.

In particular, the protective effect of ASC toward lipid peroxidation could be indirect and dependent on the synergy that ASC shows with endogenous vitamin E. In fact, the membrane-located vitamin E can reduce products of lipid oxidations; then the oxidized form of vitamin E can be rapidly reduced back by ASC in a non-enzymatic reaction [44].

Various studies showed that depletion of endogenous GSH was correlated to aging and oxidative diseases and consequently an increase in GSH intake has been proposed to promote health and wellbeing [5,45,46]. However, a low protective effect of GSH has been registered in the experimental model system analyzed in this study. This could be dependent on the fact that GSH protection might be more effective in preventing protein oxidation rather than lipoperoxidation. Consistently, glutathionylation represents a redox-dependent post-translational modification catalyzed by GSH transferase and it has been suggested as a mechanism able to mask functional thiolic group, preventing their oxidation in stressing conditions [47,48]. It has been also reported that protein glutathionylation increases under oxidative conditions with the level of glutathionylated proteins being correlated to some pathological frameworks [4]. This suggests a signaling role of this GSH- dependent post-translational mechanism with different downstream effects mainly dependent on the proteins involved in the process. Thus, the capability of GSH supplementation to really promote health remains a complex matter.

In RBC membranes, the oxidation of protein thiols has been correlated with changes in micro-elasticity and functionality of the membrane [49]. The oxidation of thiolic groups could also be an indirect effect of MDA formation under stress, being this aldehyde able to interact with proteins as it has been demonstrated in atherosclerotic processes [50]. Thus, the oxidative status of membrane proteins can indirectly reflect MDA accumulation under stress. For validating this hypothesis, the RBC membrane model here used could be optimized for studying the oxidation level of the proteins located in the membrane under UV injury in presence or absence of putative protective metabolites. This would increase the applicability of the method to a number of food matrices and antioxidant compounds showing different protective attitude toward specific class of biological molecules.

## 4. Conclusions

In this study, it is pointed out that the protective nature of different pools of phenolic compounds from food matrices and phytochemicals against oxidative stimuli cannot be correctly evaluated by in vitro antioxidant chemical tests. For this reason, an experimental model based on RBC membranes has been employed for the assessment of the capability of different plant extracts and purified molecules to prevent oxidative damage in a biological context. Data here presented also support that food complex matrices are more effective in preventing oxidative damages at biological level than pure phytochemicals, even if for these latter chemical antioxidant activity was generally higher than that observed in plant extracts. Moreover, the applied experimental model is a useful tool to measure the protective effects of various metabolites, including those in complex herbal extracts, showing chemical scavenging capability. Further studies are encouraged in order to employ RBC membranes as a biological tool for testing the protective effect of antioxidants showing different chemical characteristics toward multiple targets of oxidation.

## Figures and Tables

**Table 1:** TEAC reactivity of antioxidant standards. The ABTS scavenging activity of known antioxidants have been determined by TEAC assay and expressed as Trolox equivalent µmol per mg. Values are means ± SD of three replicates. Different letters (a), (b), (c), (d), (e) indicate a statistical difference (*p* < 0.05) according to ANOVA.

Antioxidant Standard	Total Antioxidant Capacity (µmol TE/mg)
Caffeic Acid	12.3 ± 0.3 (b)
Gallic Acid	61.8 ± 12.9 (a)
Protocatechuic acid	7.1 ± 0.4 (d)
Vanillic acid	8.7 ± 2.1 (cd)
Resveratrol	10.5 ± 1.9 (bc)
Ellagic acid	9.4 ± 0.7 (c)
ASC	6.3 ± 0.2 (e)
GSH	9.2 ± 0.5 (c)

**Table 2:** Protective effect of extracts and single antioxidants toward UV-induced oxidative stress in RBC membranes. The protective capacity of the different samples is expressed as IC_50_ (IC = inhibiting concentration), i.e., as sample concentration (µg/mL) that determines 50% of inhibition of MDA release induced by radiation stress. Values are means ± SD of three replicates. Different letters (a), (b), (c), (d) indicate a statistical difference (*p* < 0.05) according to ANOVA.

Extracts	IC_50_ (µg/mL)
ITA-seeds	39.5 ± 6.8 (a)
POM-mesocarp	155 ± 17.9 (b)
ROSE-leaves	169 ± 4.9 (b)
Vineatrol	193 ± 1.1 (b)
OLIVE-water	396 ± 66.4 (c)
**Antioxidant standards**	**IC_50_ (µg/mL)**
Protocatechuic acid	25 ± 2.0 (c)
Caffeic acid	44 ± 11.0 (b)
Gallic acid	64 ± 10.0 (b)
Resveratrol	12 ± 8.0 (d)
Ellagic acid	45 ± 10.0 (b)
ASC	60 ± 7.0 (b)
GSH	2225.9 ± 172.3 (a)
Vanillic acid	nd

## Data Availability

Data is contained within the article and supplementary material.

## Supplementary Materials

The following are available online at https://www.mdpi.com/article/10.3390/antiox10071136/s1, Figure S1: Time dependent RBC membranes’ lipid peroxidation.
