# Supplementary material for: Comparison between In Vitro Chemical and Ex Vivo Biological Assays to Evaluate Antioxidant Capacity of Botanical Extracts
Source: Antioxidants (Basel). 2021 Jul 17;10(7):1136. doi: 10.3390/antiox10071136 (PMC8301118; doi:10.3390/antiox10071136)
Supplement: Supplementary file 1 [file antioxidants-10-01136-s001.zip › antioxidants-1268641-supplementary.pdf]

SUPPLEMENTARY DATA

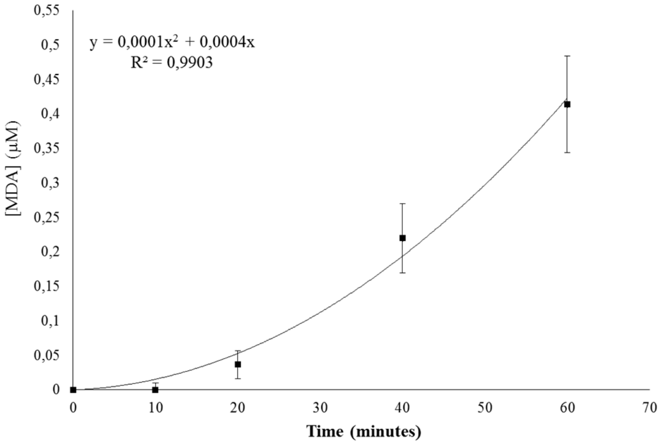

Figure S1. Time dependent RBC membranes' lipid peroxidation. Lipid peroxidation was determined through the TBARS assay. $MDA(TBA)_2$ adduct concentration (μM) is reported.
